# Health care pathways and expert patients: Do they improve outcomes?/Rutas asistenciales y paciente experto: ¿mejoran resultados?

**Published:** 2012-05-29

**Authors:** M. Sans Corrales, L. Gardeñes Morón, C. Moliner Molins, I. Campama Tutusaus, S. Pérez García, M. Rozas Martínez

**Affiliations:** GP, El Castell Primary Care Team [PCT], and Lecturer in the Department of Clinical Sciences, Bellvitge Hospital, University of Barcelona, Barcelona, Spain; GP, El Castell PCT, Barcelona, Spain; GP, El Castell PCT, Barcelona, Spain; RN, El Castell PCT, Barcelona, Spain; GP, El Castell PCT, Barcelona, Spain; GP, El Castell PCT, Barcelona, Spain

**Keywords:** shared care, continuum of care, diabetes mellitus, heart failure, chronic obstructive pulmonary disease, atención compartida, continumm asistencial, DM, IC, EPOC

## Introduction

One of the greatest challenges for health systems now and in the near future is caring for people with chronic conditions [1]. This is going to involve not only providing specific solutions for various groups of patients, but also working and, in particular, tackling challenging situations in a different way. Any one organisation or professional group alone cannot give a ‘magical’ or ‘universal’ answer to this problem. The approach must change, be multidisciplinary and subsidiary. It requires settings to be developed in which organisations and professionals not just fulfil their duties but do so in a cooperative, integrated manner, in an organisational model that is different from the current one [2].

In this context, in a territory of 124.65 km^2^, with catchment population of 180,000, one secondary care hospital and seven primary care teams (PCT), one of which has formed a consortium with the local council, a joint project was designed to approach the most common chronic diseases. The aims are to improve the quality and continuum of healthcare provided, holding a first meeting between healthcare levels, primary care (PC) and specialised care (SC), in order to establish care pathways (CPs) for the following diseases: type 2 diabetes mellitus (DM2), heart failure (HF), and chronic obstructive pulmonary disease (COPD).

The overall objective of the project team was to improve the follow-up of chronic patients, using the CPs and involving nurses in the education of the COPD and HF patients, through an expert patient (EP) programme of the Catalan Institute of Health (ICS), that promotes self-care by exchanging knowledge between the EP and other participants with COPD and HF.

## Description of the intervention

Working groups were established between professionals in SC and PC (a GP and a PC nurse). During 2010, these groups had regular meetings with others involved in the care pathways to establish the action protocols, based on clinical practice guidelines, and these were then presented to the rest of the PC and SC professionals.

In the team, the following specific objectives were set: for **COPD**, to increase the percentage of patients who, in the previous two years, have undergone spirometry and have been advised to give up smoking, and to decrease the number of admissions for exacerbations; **HF**: to increase the percentage of patients whose BMI has been measured in the previous year and whose status has been categorised according to the NYHA (New York Heart Association) functional classification, to visit patients under home care 2–3 times per year, to achieve appropriate prescription of beta-blockers, to improve the monitoring of blood pressure (BP), and to decrease the number of admissions for decompensation; and for **DM**, to increase the percentage of patients with cardiovascular risk assessments, with two measurements of weight and glycated haemoglobin (HbA1C )per year, and with acceptable control of HbA1C, blood pressure and BMI.

For this purpose, meetings were held between the members of the team in charge of the pathways, the management and the other health professionals involved. The aim of the these meetings being to make everyone aware of the pathways, provide disease updates and clinical follow-up; in which lists of patients poorly controlled according to the assessment indicators and incidents were distributed.

**Figure 1.**
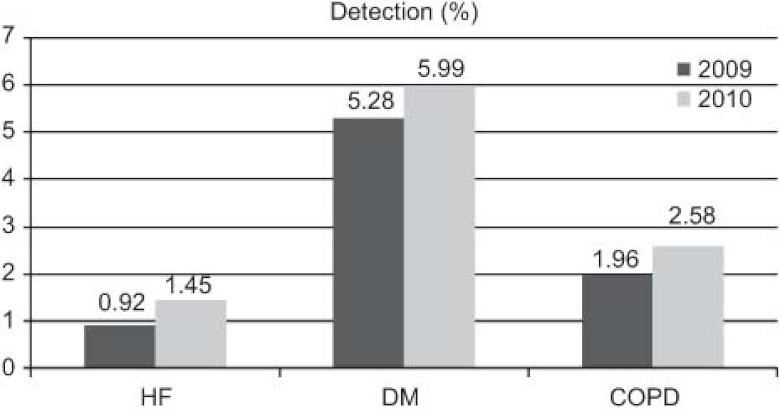
••••

**Figure 2.**
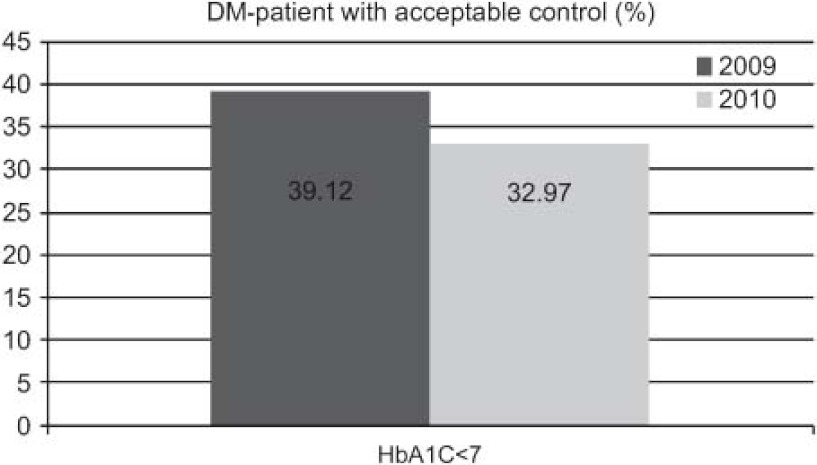
••••

**Figure 3.**
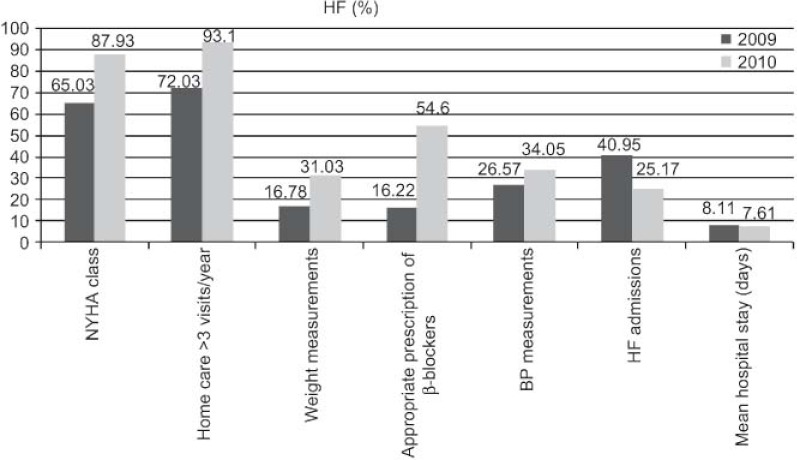
••••

**Figure 4.**
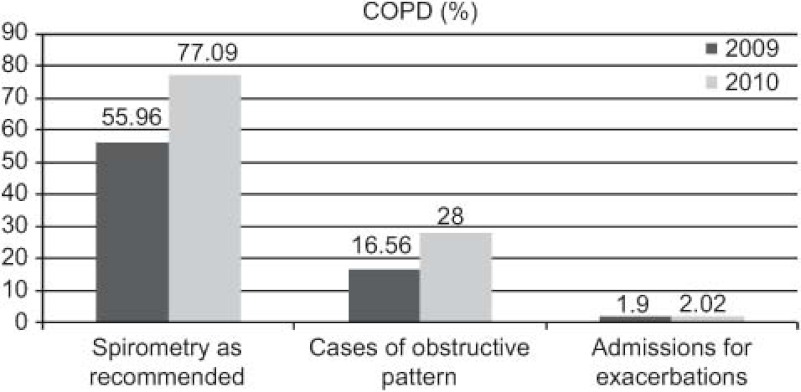
••••

**Figure 5.**
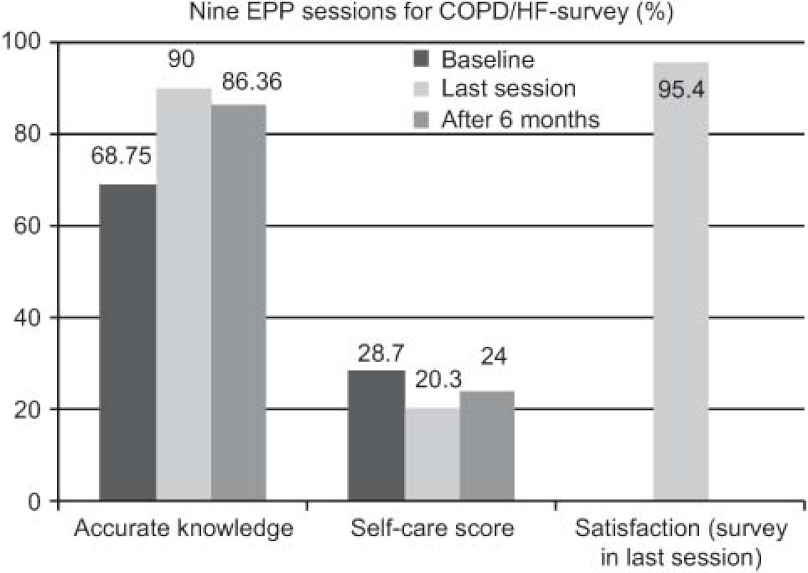
••••

The outcomes achieved (Figures 1–5) are compared to those of 2009 (before the establishment of the care pathways).

- Increase in detection: 89 new cases HF (0.92–1.45%), 211 of DM (5.28–5.99%), and 71 of COPD (1.96–2.58%).
- DM: increases in 6-month repeat assessment of HbA1C levels (30.05–39.60%), REGICOR score (62–83.85%; p<0.0001), weight (44.66–50.76%; p<0.0001), BP (59.59–64.08%; p<0.025) and BMI (41.63–47.37%; p<0.01), as well as a decrease in the percentage of patients with acceptable control (HbA1C<7) (39.12–32.97%; p<0.01).
- COPD: improvement in use of spirometry (55.96–77.09%) and increase in the rate of cases found with an obstructive pattern (16.56–28%; p<0.01), and increase in the percentage of patients advised in the previous 2 years to stop smoking from 33% to 68.83%, while there was also an increase in emergency admissions for exacerbations (1.9–2.02%) but this was not statistically significant.
- HF: increases in patients with NYHA functional class recorded (65.03–87.93%; p<0.001), with weight measurements (16.78–45.52%; p<0.001), receiving at least three visits/per year from a PC nurse (72.03–93.10%; p<0.001), with prescriptions of beta-blockers reviewed/modified (16.22–54.60%; p<0.001), and with BP measurements (26.57–34.05%), while there were decreases in the number of hospital admissions for decompensation (40.95–25.17%) and the mean length of hospital stay (8.11–7.61 days).

Nine sessions of the EP programme were carried out for COPD and HF. Questionnaires were completed at baseline, after 6 months and at the end of the intervention: accurate knowledge 68.75, 86.36 and 90.0%, respectively; European Heart Failure Self-care Behaviour scale: 28.7, 24.0 and 20.3%, respectively; and satisfaction questionnaire in the last session: 95.4%.

## Discussion

Since the establishment of disease-specific care pathways, there has been an increase in the rates of detection and monitoring of the corresponding three diseases.

With regards to DM, it should also be highlighted that there was an increase in compliance with the protocol agreed between PC and SC, improving the 6-monthly measurements and recording of glycated haemoglobin levels, weight, and blood pressure, as well as calculation of BMI and assessment of cardiovascular risk. On the other hand, it may seem surprising that there was no increase in the percentage of patients with an acceptable control of their condition; indeed, the rate decreased. We attribute this to the increase in detection of new cases since the establishment of the care pathway.

As for HF, there was a notable the reduction in the number of hospital admissions for HF as well as the average hospital stay, which may be explained due to the increase in follow-up home visits by the end of the year, better control of blood pressure, spectacular increase in the review and modification of treatment with beta-blockers and monitoring of weight as an indicator of possible clinical worsening.

In the case of COPD, we highlight the increase in the rates of spirometry tests (in line with recommendations) and detection of cases with an obstructive pattern, although the rate of emergency admissions for exacerbations did not significantly change.

In addition, together with the care pathways, the EP programme of the ICS has had an impact: improving patient knowledge on their own disease and increasing self-care (lower scores on the European self-care scale indicating better self-care). In turn, these two factors may also have helped achieve the reduction in the rate of the hospital admissions for HF. Finally, there was a notably high level of satisfaction among patients who participated in the EP programme.

## Conclusions

Care pathways are a valid tool for achieving coordination between levels of care and increasing the involvement of professionals, leading to a clear improvement in process indicators. The EP programme does help patients increase their understanding of their condition and enable them to improve their quality of life.

## Conference abstract Spanish

## Introducción

La atención a personas con problemas crónicos constituye uno de los retos más importantes que debe afrontar el sistema de salud en los próximos años [1]. No se trata sólo de aportar soluciones específicas para diferentes grupos de pacientes, sino de trabajar y abordar las situaciones de manera distinta. Difícilmente una sola organización o un solo grupo profesional pueden dar una respuesta “mágica” y “universal” a estas nuevas situaciones. El abordaje debe ser diferente, multidisciplinar y subsidiario, exige escenarios donde cada organización y profesional haga lo que le toca hacer pero se haga también de manera cooperativa, integradamente, en un modelo organizativo distinto al actual [2].

De ahí que en un territorio de 124,65 km^2^, 180.000 habitantes, 7 Equipos Atención Primaria (EAP), 1 Hospital de segundo nivel y 1 EAP consorciado con el Ayuntamiento, se diseñó un proyecto común de abordaje a la patología crónica más prevalente, para mejorar la calidad y el continuum asistencial, celebrando una primera reunión entre niveles asistenciales atención primaria (AP)- atención especializada (AE), con el objetivo de elaborar rutas asistenciales (RA) de las siguientes patologías: diabetes mellitas tipo 2 (DM2), insuficiencia cardíaca (IC), enfermedad pulmonar obstructiva crónica (EPOC).

Así mismo, el equipo tuvo como objetivo mejorar el seguimiento de los pacientes crónicos, a partir de las RA e implicar a enfermería en la educación del paciente EPOC e IC, mediante el programa paciente experto (PE) del “Institut Català de la Salut” que potencia el autocuidado mediante el intercambio de conocimientos entre un Paciente Experto (PE) y el resto de participantes con EPOC o IC.

## Descripción de la intervención

Se organizaron grupos de trabajo con profesionales de AE y AP (un médico y un diplomado en enfermería por EAP). Durante el 2010, dichos grupos realizaron reuniones periódicas con el resto de referentes de rutas para elaborar los protocolos de actuación, basados en guías de práctica clínica; los cuales fueron presentados al resto de profesionales de AP y AE.

En el equipo, se fijaron los siguientes objetivos: **EPOC**: aumentar porcentaje de pacientes con espirometría últimos 2 años, aumentar porcentaje pacientes con consejo antitabaco últimos 2 años, disminuir el número de ingresos por reagudizaciones;** IC**: aumentar porcentaje pacientes con registro de Indice de Masa Corporal (IMC) último año, porcentaje pacientes con registro NYHA (New York Heart Association), realizar 2–3 visitas año pacientes en atención domiciliaria, adecuación de tratamiento beta-bloqueante, control tensión arterial, disminuir número ingresos por reagudización; **DM**: aumentar porcentaje pacientes con registro riesgo cardiovascular, 2 determinaciones de peso y HbA1C por año, control adecuado HbA1C y tensión e IMC.

Para ello, se realizaron trimestralmente con los referentes de las diferentes rutas del equipo, la dirección y el resto de profesionales sanitarios, sesiones de presentación de las rutas, de actualizaciones de las patologías y de seguimiento clínico; en las cuales se distribuían listados de pacientes mal controlados según indicadores de evaluación y recogida incidencias.

**Figura 6.**
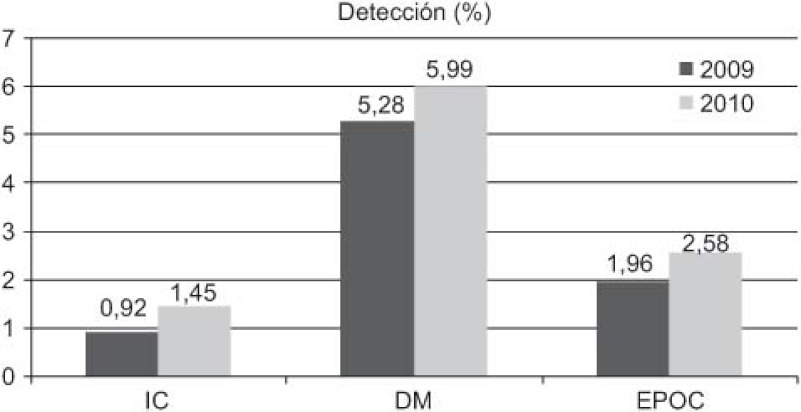
••••

**Figura 7.**
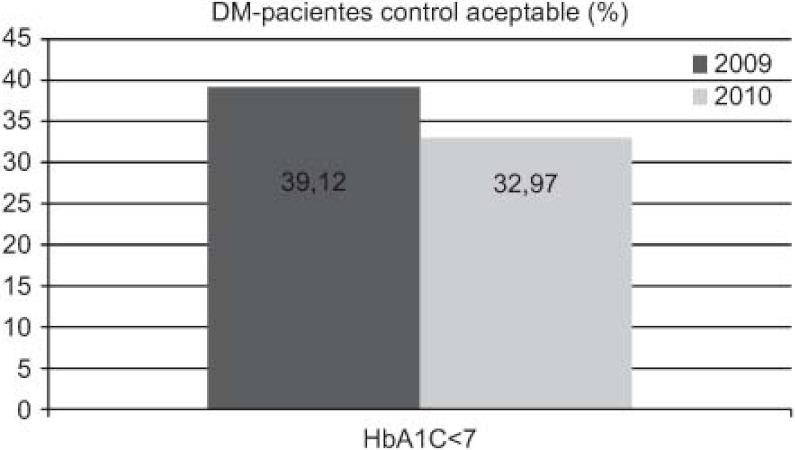
••••

**Figura 8.**
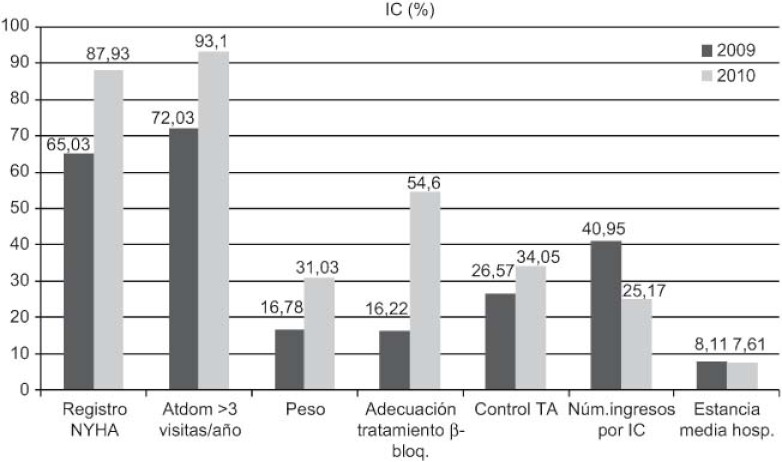
••••

**Figura 9.**
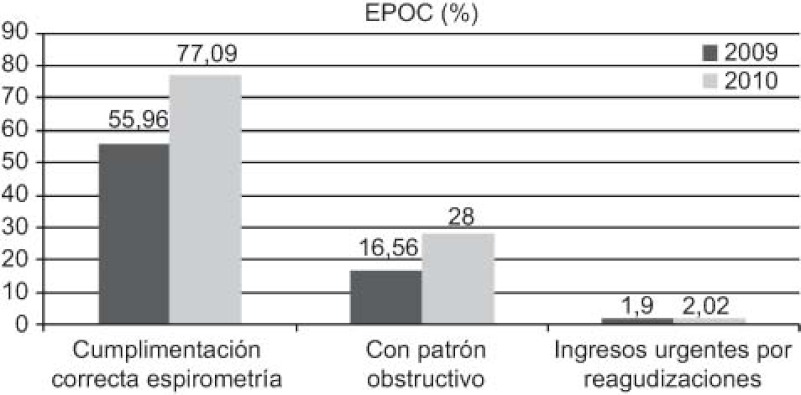
••••

**Figura 10.**
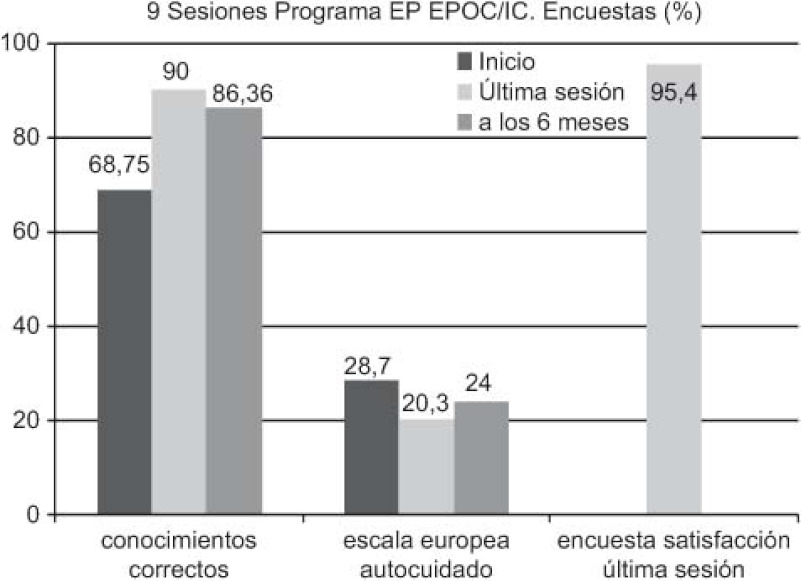
••••

Los resultados obtenidos (Figuras 6–10), se presentan en comparación a los del 2009 (sin rutas):

- Aumenta detección: 89 nuevos casos IC (0,92–1,45%), 211 DM (5,28–5,99%), 71 EPOC (1,96–2,58%).
- DM: aumenta controles semestrales hemoglobina glicada (HbA1C) (30,05–39,60%), REGICOR (62–83,85%; p<0,0001), peso (44,66–50,76%; p<0,0001), TA (59,59–64,08%; p<0,025), IMC (41,63–47,37%; p<0,01), disminuye porcentaje pacientes control aceptable (HbA1C<7) (39,12–32,97%; p<0,01).
- EPOC: mejora cumplimentación espirometría (55,96–77,09%) y de ésta con patrón obstructivo (16,56–28%; p<0,01), aumentan los ingresos urgentes por reagudizaciones (1,9–2,02%; no significativo), aumenta el porcentaje de número de pacientes con consejo antitabaco del 33% al 68,83% en los últimos 2 años.
- IC: aumenta registro clasificación funcional NYHA (65,03–87,93%; p<0,001), peso (16,78–45,52%; p<0,001), pacientes con mínimo de 3 visitas/año por EAP (72,03–93,10%; p<0,001) y adecuación tratamiento betabloqueante (16,22–54,60%; p<0,001), control tensión arterial (TA) (26,57–34,05%), disminuye número ingresos hospitalarios por descompensación (40,95–25,17%), disminuyendo estancia media hospitalaria (8,11–7,61 días).

Realizadas nueve sesiones del programa PE para EPOC, IC. Encuestas al inicio, a los 6 meses y en la última sesión: conocimientos correctos 68,75; 86,36 y 90%; escala europea autocuidado (*European Heart Failure Self-care Behaviour scale*28,7; 24 y 20,3% y encuesta satisfacción última sesión: 95,4%.

## Discusión

Desde la instauración de las RA cabe destacar el aumento de la detección y registro de las tres patologías para las cuales se han establecido las rutas.

En cuanto a la DM, también hay que remarcar que aumenta la cumplimentación del protocolo consensuado entre AP y AE mejorando los registros semestrales de hemoglobina glicada, cálculo del riesgo cardiovascular, peso, tensión arterial e índice de masa corporal, pero podría sorprender que no conlleve un incremento del porcentaje de pacientes con control aceptable. Esta disminución del porcentaje la atribuimos al aumento de detección de casos nuevos desde la implantación de la ruta asistencial.

En referencia a la IC, llama la atención la reducción del número de ingresos hospitalarios por IC y también la estancia media hospitalaria, que se podría explicar por el incremento de visitas domiciliarias de seguimiento al año, el mejor control de la TA, el espectacular incremento de la adecuación del tratamiento betabloqueante y el seguimiento del peso como indicador de descompensación.

Respecto a la EPOC, destaca el incremento de la realización correcta de espirometría y detección de patrón obstructivo, aunque se mantiene prácticamente igual el número de ingresos urgentes por reagudizaciones.

Asimismo y añadido a las RA, el programa PE del Institut Català de la Salut también ha influido en la mejora de los conocimientos de los pacientes sobre su propia enfermedad, incrementando el autocuidado, dado que una puntuación menor en la escala europea de autocuidado significa justamente, a la inversa, mayor autocuidado. Estos dos factores también pueden influir en la reducción del porcentaje de ingresos hospitalarios. También cabe destacar el alto grado de satisfacción de los pacientes que participaron en el programa PE.

## Conclusiones

Las RA son una herramienta válida para la coordinación entre niveles asistenciales y la implicación de los profesionales, produciendo clara mejora en indicadores de procesos. El Programa PE ayuda a los pacientes a entender mejor su enfermedad y les permite conseguir mayor calidad de vida.
